# Akt-Dependent Glycolysis-Driven Lipogenesis Supports Proliferation and Survival of Human Pulmonary Arterial Smooth Muscle Cells in Pulmonary Hypertension

**DOI:** 10.3389/fmed.2022.886868

**Published:** 2022-06-28

**Authors:** Lifeng Jiang, Dmitry A. Goncharov, Yuanjun Shen, Derek Lin, Baojun Chang, Andressa Pena, Horace DeLisser, Elena A. Goncharova, Tatiana V. Kudryashova

**Affiliations:** ^1^Lung Center, Division of Pulmonary, Critical Care and Sleep Medicine, Department of Internal Medicine, School of Medicine, University of California, Davis, Davis, CA, United States; ^2^Pittsburgh Heart, Lung, and Blood Vascular Medicine Institute, University of Pittsburgh, Pittsburgh, PA, United States; ^3^Department of Medicine, Perelman School of Medicine, University of Pennsylvania, Philadelphia, PA, United States

**Keywords:** pulmonary arterial hypertension, lipogenesis, vascular smooth muscle, proliferation, apoptosis, JNK, SIRT7, Akt

## Abstract

Hyper-proliferation of pulmonary arterial vascular smooth muscle cells (PAVSMC) is an important pathological component of pulmonary vascular remodeling in pulmonary arterial hypertension (PAH). Lipogenesis is linked to numerous proliferative diseases, but its role in PAVSMC proliferation in PAH remains to be elucidated. We found that early-passage human PAH PAVSMC had significant up-regulation of key fatty acids synthesis enzymes ATP-citrate lyase (ACLY), acetyl-CoA carboxylase (ACC), and fatty acid synthase (FASN), and increased unstimulated proliferation compared to control human PAVSMC. Treatment with an allosteric ACC inhibitor 5-tetradecyloxy-2-furoic acid (TOFA) significantly decreased proliferation and induced apoptosis of human PAH PAVSMC. Intracellular lipid content and proliferation of PAH PAVSMC were not reduced by incubation in lipid-depleted media but suppressed by a non-metabolizable analog of glucose 2-Deoxy-D-glucose (2-DG) and partially restored by addition of pyruvate. Protein kinase Akt was upregulated in human PAH PAVSMC in a sirtuin 7 (SIRT7)- and c-Jun N-terminal kinase (JNK)-dependent manner. Pharmacological inhibition of Akt down-regulated ACLY and ACC, significantly reduced intracellular lipid content, inhibited proliferation and induced apoptosis of human PAH PAVSMC. Taken together, these data demonstrate that human PAH PAVSMC have up-regulated lipogenesis, which is supported in an Akt- and glycolysis-dependent manner and is required for increased proliferation and survival. Our data suggest that there is a mechanistic link between glycolysis, lipogenesis, and the proliferation of human PAH PAVSMC and call for further studies to determine the potential attractiveness of a SIRT7/JNK-Akt-lipogenesis axis as a target pathway to inhibit PAVSMC hyper-proliferation in PAH.

## Introduction

Pulmonary arterial hypertension (PAH) is a devastating progressive disease leading to a deteriorating quality of life and high morbidity and mortality rates (1–3). Continuous vasoconstriction and excessive remodeling of pulmonary arteries (PA) cause an increase in pulmonary arterial pressure and pulmonary vascular resistance, leading to elevated right ventricular (RV) afterload and ultimately right heart failure and death (4, 5). At present, available vasodilatory therapies do not stop disease progression, and currently there are no vascular remodeling-focused therapies available for PAH patients (4, 6). All three layers of small pulmonary arteries—intima, media and adventitia—contribute to pulmonary vascular remodeling (7, 8). One of the important features of pulmonary vascular remodeling is the increased proliferation and survival of pulmonary arterial vascular smooth muscle cells (PAVSMC) (9, 10) in the medial layer of small muscular PAs (11). Over the last decades significant progress was achieved in dissecting the signaling molecules and pathways supporting the pathological pro-proliferative/pro-survival nature of PAVSMC in PAH. However, the underlying mechanisms are still not completely understood.

We and others previously demonstrated that PAH PAVSMC undergo a complex metabolic reprogramming required to maintain energy consuming pro-proliferative phenotype (9, 10, 12, 13). In contrast to non-diseased cells, PAH PAVSMC demonstrate increased ATP generation, proliferation and survival which depend predominantly on glycolytic metabolism (10) and undergo a metabolic shift from mitochondrial oxidative phosphorylation to glycolysis, similar to the “Warburg effect” in cancer cells (9, 10, 14). Multiple crucial metabolic regulators and enzymes, including AMP-activated protein kinase (AMPK), mechanistic target of rapamycin (mTOR) complex 2 (mTORC2) (10), hypoxia-induced factor (HIF) 1α (9), nuclear factor of activated T-cells (NFAT) (15), peroxisome proliferator-activated receptor (PPAR)γ (16), pyruvate dehydrogenase (PDH) (17), PDH kinase (PDK) (18), 6-phosphofructo-2-kinase/fructose-2,6-bisphosphatase 3 (PFKFB3) (19), pyruvate carboxylase (PC) (20), and enolase (21), support this glycolytic shift in PAH PAVSMC (9, 10, 22). A stable isotope metabolomics-based study confirmed that PAH PAVSMC have increased glucose uptake and utilization by glycolysis and the pentose shunt, but intriguingly no changes in fatty acid or glutamine uptake or utilization were detected (23).

Besides increased ATP levels, hyper-proliferative cells require increased amounts of intracellular essential “building blocks” such as lipids, proteins, and nucleic acids (24). The fact that highly proliferative PAH PAVSMC do not demonstrate increased fatty acid uptake (23) indicates that lipid metabolism in these cells might be re-organized to produce and accumulate the required amount of lipids internally. Indeed, several lines of evidence indicate that lipid metabolism is deregulated in PAH PAVSMC (25). RNAseq-based analysis identified up-regulation of fatty acid biosynthesis and metabolism pathways in isolated human PAH PASMC (26). Up-regulation of a key enzyme in fatty acid synthesis, fatty acid synthase (FASN) (27), and a key rate-limiting enzyme of mitochondrial fatty acid β-oxidation, carnitine palmitoyltransferase (CPT) (28), was reported in PAVSMC in rats with monocrotaline (MCT)-induced PH. Interestingly, a similar metabolic adaptation to satisfy high lipids demand is observed in most cancer cells, which have elevated endogenous fatty acid synthesis supported by the increased glycolysis and an increased ability to synthesize lipids (29, 30). *De novo* fatty acid synthesis makes a major contribution to the intracellular fatty acid pool in tumor cells (31) suggesting that PAVSMC in PAH might use similar strategies to support their highly proliferative phenotype. However, in contrast to the role of lipid metabolism in right ventricle (RV) function in PAH (25), the potential role of the lipogenic process and underlying mechanisms in PAH PAVSMC require further investigation.

In this study, we aimed to evaluate the status and role of lipogenesis in PAH PAVSMC proliferation and survival. Our data demonstrate that the pro-proliferative/pro-survival phenotype of PAVSMC in PAH is supported by glycolysis-dependent *de novo* lipid synthesis and suggest the potential role of Sirtuin 7 (SIRT7)- c-Jun N-terminal kinase (JNK)-Akt axis as a regulator of lipogenesis and a potential molecular target for anti-remodeling therapy.

## Materials and Methods

### Cell Culture

Early-passage (3–8 passage) human PAVSMC isolated from small (≤1 mm outer diameter) PAs of patients with PAH and non-diseased subjects were provided by UC Davis Lung Center Pulmonary Vascular Disease Program human specimens biobank, University of Pittsburgh Vascular Medicine Institute Cell Processing Core, and the Pulmonary Hypertension Breakthrough Initiative (PHBI) under approved protocols in accordance with Institutional Review Board (IRB) and Committee for Oversight of Research and Clinical Training Involving Decedents (*CORID*) policies. Cells isolation, characterization and maintenance were performed under PHBI-approved protocols as described previously (10, 32). Cells were maintained in complete PromoCell Smooth Muscle Cell Growth Medium 2 with SupplementPack and Antibiotic-Antimycotic. Before experiments, cells were incubated for 24–48 h in basal media supplemented with 0.1% bovine serum albumin (BSA) if not stated otherwise. For functional experiments (Ki-67, cell counts, TUNEL) a minimum of three technical replicas was performed within one experiment, minimum of 100 cells/subject was analyzed.

### Exogenous Lipids Removal

To achieve the lipid-deprived cell culture condition, cell culture grade fetal bovine serum (FBS) and BSA were delipidated using Cleanascite*™* Lipid Removal Reagent (Biotech Support Group; Monmouth Junction, NJ, United States) according to the manufacturer’s protocol with modification. Briefly, Cleanascite*™* was added to the FBS or BSA (1:4 volume ratio). The mixture was incubated for four hours at 4°C with gentle shaking and centrifuged at 16,000 *g* for 10 min. Supernatant was collected, then a second dose of Cleanascite*™* Lipid Removal Reagent was added (1:4 volume ratio), and incubation and centrifugation steps were repeated as described above. The supernatants, consisting of lipid depleted FBS or BSA, were then used as cell culture media supplements to prepare lipid-depleted media.

### Intracellular Lipid Detection

Intracellular lipid detection was performed using a fluorescent probe for lipid droplets BODIPY 493/503 as previously described in Qiu et al. (33) with modifications. Briefly, cells were washed twice with PBS and then incubated with 2 μM BODIPY 493/503 staining solution (#D3922 Thermo Fisher Scientific, Chicago, IL, United States) for 15 min at 37°C. Then cells were fixed in 4% paraformaldehyde/phosphate-buffered saline (PBS), followed by DAPI staining to detect nuclei. Immunostaining was visualized and images were taken using a Keyence BZ-X800 (Keyence Corporation of America, Itasca, IL, United States) and Zeiss LSM700 confocal microscope (White Plains, NY, United States). A minimum of 100 cells per condition was analyzed.

### Cell Proliferation Assay

Cell proliferation was assessed using Ki-67 immunostaining as described previously (10, 32, 34). Briefly, cells were washed with PBS, fixed in 4% paraformaldehyde/PBS, and permeabilized using Triton X-100/PBS solution. Then cells were incubated in blocking solution (2% BSA/PBS), followed by overnight incubation with primary anti-Ki-67 antibody (#9129, Cell Signaling, Danvers, MA, United States) in blocking solution. Next day, the slides were washed with PBS, followed by incubation with secondary chicken anti-rabbit IgG (H + L) Alexa Fluor 594 antibody (#A-21442, Invitrogen, Waltham, MA, United States) and 4′,6-diamidino-2-phenylindole (DAPI) to detect nuclei. Images were taken using a Keyence BZ-X800 microscope.

### Cell Growth Assay

Cell growth analysis was performed as described previously (10, 32, 34). Briefly, 300,000 cells per well were plated in a six well plate in cell culture media supplemented with 5% FBS. Forty-eight hours later, when cells attached and spread (day 0), the media was changed to 0.1% BSA with or without lipids and incubated for six more days (media was renewed every 48 h). Then, cell counting was performed using the Countess II FL Automated Cell Counter (Thermo Fisher Scientific, Waltham, MA, United States) according to manufacturer’s protocol.

### Apoptosis Analysis

Apoptosis analysis was performed using the *In situ* Cell Death Detection Kit (Roche, Nutley, NJ, United States) based on terminal deoxynucleotidyltransferase-mediated dUTP-biotin nick end labeling (TUNEL) technology following the manufacturer’s protocol.

### Immunoblot Analysis

Immunoblot analysis was performed as described previously (10, 32, 34). Antibodies for ACLY (#4332), P-S79-ACC (#11818), ACC (#3676), FASN (#3180), P-S473-Akt (#4060), P-T450-Akt (#12178), Akt (#9272), PThr183/Tyr185-JNK (#4668), JNK (#9252), α/β-Tubulin (#2148), CPT1A (#97361), hexokinase II (HKII) (#2867), phosphofructokinase (PFKP) (#8164) were purchased from Cell Signaling (Danvers, MA, United States). The antibody for P-S455-ACLY (#PA5-97395) was purchased from Thermo Fisher Scientific (Chicago, IL, United States). The antibody for malonyl CoA decarboxylase (MLYCD) (#15265-1-AP) were purchased from Proteintech (Rosemont, IL, United States). Secondary HRP conjugated anti-mouse antibody (ab205719) was purchased from Abcam (Boston, MA, United States), secondary HRP-conjugated anti-rabbit antibody (#7074) was purchased from Cell Signaling (Danvers, MA, United States).

### Inhibitors and Activators

2-Deoxy-D-glucose (2-DG, D6134), IL-6 (I1395), PDGF-BB (GF149), and Akt inhibitor VIII (SIAL-124018) were purchased from Millipore Sigma (St. Louis, MO, United States), 5-tetradecyloxy-2-furoic acid (TOFA, sc-200653) was purchased from Santa Cruz Biotechnology (Dallas, TX, United States), JNK inhibitor (bentamapimod, HY-14761) was purchased from MedChemExpress (Monmouth Junction, NJ, United States).

### Lactate Assay

To measure intracellular lactate amount, the lactate assay kit (#MAK064, Millipore Sigma) was used according to the manufacturer protocol with modifications. Briefly, 2 × 10^6^ cell were homogenized in 200 μL of lactate assay buffer and centrifuged at 21,000 *g* for 5 min to remove cell debris. The supernatant was deproteinized with a 10 kDa MWCO spin filter (#UFC5003, Millipore Sigma) to remove lactate dehydrogenase, and the lactate assay was performed. Protein concentrations were determined in the supernatants before deproteinization using the BCA protein assay kit (#23227, Thermo Fisher Scientific) and lactate content was normalized to the amount of total protein used for the assay.

### Data Analysis

Immunoblots, BODIPY 493/503, Ki-67 and apoptosis assays were analyzed using ImageJ (NIH, Bethesda, MD, United States), StatView (SAS Institute, Cary, NC, United States) and GraphPad Prizm 9.02 (GraphPad Software, San Diego, CA, United States). Immunocytochemical analyses were detected and captured using the Keyence BZ-X800 system and software (Keyence Corporation of America, Itasca, IL, United States) and Zeiss LSM700 confocal microscope and software (ZEN, 2009; White Plains, NY, United States). Statistical comparisons between the two groups were performed by Mann–Whitney *U* test. Statistical comparisons among three or more groups were performed by the Kruskal–Wallis rank test with Dunn pairwise comparison *post hoc* test. Statistical significance was defined as *p* ≤ 0.05.

## Results

### Increased Lipid Synthesis Is Required for Hyper-Proliferation and Survival of Human PAH PAVSMC

**FIGURE 1:**
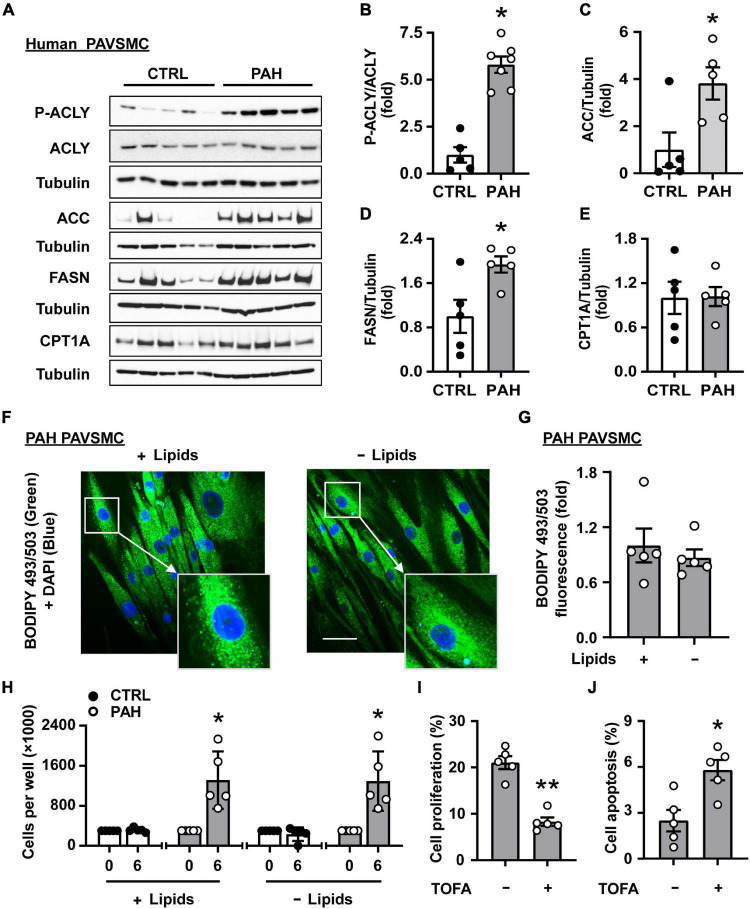
Increased lipid synthesis is required for hyper-proliferation and survival of human PAH PAVSMC. **(A–E)** Early passage distal primary human PAVSMC from non-diseased (CTRL) and PAH subjects were serum-deprived for 48 hours and subjected to immunoblot analysis to detect indicated proteins. *n* = 5 (CTRL), *n* = 7 (PAH for P-ACLY, ACLY, see Supplementary Figure 1A for additional immunoblots used for statistical analysis) or *n* = 5 (PAH for ACC, FASN, CPT1A). Data are means ± SE, fold to control. **(F,G)** Human PAH PAVSMC were incubated for 48 h in serum deprived media supplemented with regular (+ Lipids) or lipid-deprived (-Lipids) 0.1% BSA and subjected to fluorescent BODIPY 493/503 staining (green) to detect intracellular neutral lipids followed by DAPI (blue) staining to detect nuclei. Representative images with enlarged area **(F)** and statistical analysis **(G)** are shown. Bar equals 50 μm. Data are means ± SE from *n* = 5 subjects/group, fold to cells incubated in regular 0.1% BSA (+ Lipids) group. **(H)** Equal amount of human PAVSMC from five PAH and five control subjects were seeded to 6 well plates. 48 h later (day 0), media was changed to the serum-free media supplemented with regular (+ Lipids) or lipid-deprived (-Lipids) 0.1% BSA; six days later, cell count assay was performed. Data are means ± SE, *n* = 5 subjects/group. **(I)** PAH PAVSMC were treated with ACC inhibitor TOFA (20 μM) for 48 h followed by proliferation analysis (Ki-67). Data are means ± SE, percentage of Ki-67 positive cells/total number of cells, *n* = 5 subjects/group. **(J)** PAH PAVSMC were treated with ACC inhibitor TOFA (20 μM) for 48 h followed by apoptosis analysis. Data are means ± SE, percentage of TUNEL-positive cells/total number of cells, *n* = 5 subjects/group. *p < 0.05, **p < 0.01 by Mann–Whitney *U* test.

In order to evaluate the status of lipogenesis in PAH PAVSMC, we first tested the expression of key lipogenic enzymes driving the biosynthesis of fatty acids. Immunoblot analysis demonstrated significant increase in phosphorylated ACLY, and elevated protein levels of ACC and FASN in early-passage distal human PAH PAVSMC compared to cells from non-diseased subjects (CTRL) (Figures 1A–D and Supplementary Figure 1A). Interestingly, we detected no significant differences between non-diseased and PAH PAVSMC in protein levels of CPT1A (Figures 1A,E) and MLYCD (Supplementary Figure 1B), the regulatory enzymes in fatty acid β-oxidation and synthesis, in spite of previously reported up-regulation of CPT1A in smooth muscle cells in rats with monocrotaline-induced PH and protective effect of MLYCD deletion against development of hypoxia-induced PH in mice (28, 35). Observed up-regulation of key fatty acid synthesis enzymes suggested that *de novo* lipid synthesis is altered in PAH PAVSMC. To confirm our observations, we performed immunocytochemical detection of lipid droplets (a major storage depot for neutral lipids, primarily triglycerides) using a fluorescent probe (BODIPY 493/503) to visualize neutral lipid accumulation in PAH PAVSMC. We found that lipid accumulation detected in PAH PAVSMC was preserved in media deprived from exogenous lipids (Figures 1F,G), indicating that PAH PAVSMC have an ability to generate lipids *de novo.* Importantly, PAH PAVSMC, in contrast to control cells, demonstrated increased growth not only in serum-deprived media, but in lipid-deprived serum-deprived media (Figure 1H). Moreover, 5-tetradecyloxy-2-furoic acid (TOFA), an allosteric inhibitor of ACC, a key enzyme regulating fatty acid synthesis, significantly decreased proliferation and induced apoptosis in PAH PAVSMC (Figures 1I,J), demonstrating that lipogenesis is required for PAH PAVSMC hyper-proliferation and survival. Taken together, these data show that *de novo* lipid synthesis is up-regulated and required for maintaining the pro-proliferative/pro-survival phenotype of human PAH PAVSMC.

### Increased Lipid Synthesis in Human PAH PAVSMC Is Glucose-Dependent

**FIGURE 2:**
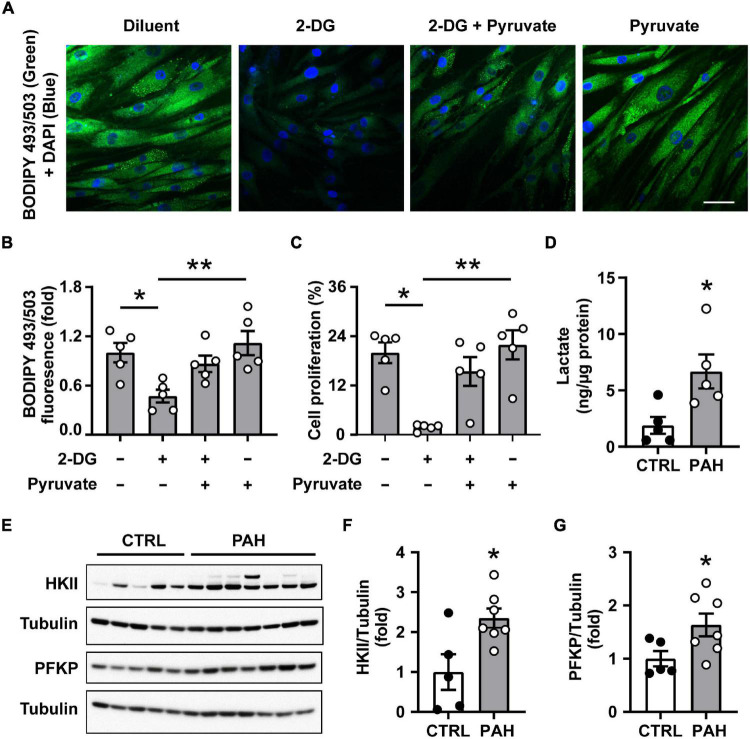
Increased lipid synthesis in human PAH PAVSMC is glucose dependent. **(A–C)** Human PAH PAVSMC were incubated in serum deprived media supplemented with 0.1% lipid-deprived BSA in presence of 2-Deoxy-D-glucose (2-DG, 100 mM) and/or pyruvate (10 mM) or vehicle (−). Forty-eight hours later neutral lipid accumulation **(A,B)** was evaluated by fluorescent BODIPY 493/503 staining (green) followed by DAPI co-staining (blue) to detect nuclei and cell proliferation (Ki-67) **(C)** was analyzed. Representative images **(A)** and statistical analysis **(B,C)** are shown. Bar equals 50 μm. Data are means ± SE, *n* = 5 subjects/group, fold to vehicle-treated group **(B)**, percentage of Ki-67 positive cells/total **(C)**, *p < 0.05, **p < 0.01 by Kruskal–Wallis rank test with Dunn pairwise comparison *post hoc* test. **(D)** Intracellular lactate levels were measured in PAVSMC from five non-diseased and five PAH subjects. **(E–G)** Early passage distal primary human PAVSMC from five non-diseased (CTRL) and seven PAH subjects were serum deprived for 48 h and subjected to immunoblot analysis to detect indicated proteins. **(D,F,G)** Data are means ± SE, fold to control **(F,G)**, *p < 0.05 by Mann–Whitney *U* test.

Since fatty acid uptake is not altered in PAH PAVSMC (23) and we found that PAH PAVSMC growth was maintained in lipid-deprived media, we hypothesized that glucose could be a potential source for *de novo* lipid synthesis in PAH. We found that intracellular lipid content in PAH PAVSMC in lipid-deprived conditions was significantly depleted by the treatment with the non-metabolizable analog of glucose 2-DG. Importantly, co-treatment with glucose metabolite pyruvate significantly attenuated 2-DG-dependent inhibition of intracellular lipid accumulation in human PAH PAVSMC (Figures 2A,B). Furthermore, 2-DG-induced inhibition of PAH PAVSMC proliferation in lipid-deprived conditions was also partially reversed by pyruvate (Figure 2C). These data demonstrate that glucose, metabolized through the glycolysis, serves as the main source for *de novo* lipid synthesis and supports lipid accumulation and increased proliferation of PAH PAVSMC. Confirming up-regulation of glycolysis, intracellular lactate levels were significantly higher in PAH PAVSMC compared to non-diseased cells (Figure 2D). Moreover, protein levels of key glycolytic enzymes PFKP and HKII were significantly higher in human PAH PAVSMC compared to controls (Figures 2E–G), and HIF1α over accumulation was detected in six out of seven PAVSMC from PAH patients compared to one out of five non-diseased (control) subjects (Supplementary Figure 2). This is in good agreement with previously published data demonstrating the importance of glycolysis for PH (19, 36, 37). Together, our data demonstrate that glycolysis-metabolized glucose serves as a main source for increased *de novo* lipid synthesis in PAH PAVSMC, supporting a pro-proliferative cell phenotype.

### Akt Supports ACLY and ACC Up-Regulation in Human PAH PAVSMC

**FIGURE 3:**
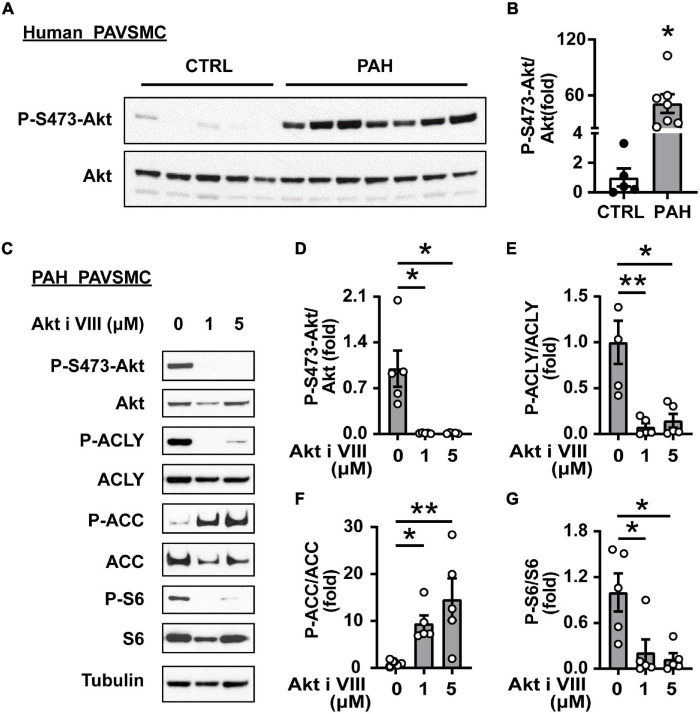
Akt supports ACLY and ACC up-regulation in human PAH PAVSMC. **(A,B)** Early passage distal human PAVSMC from five non-diseased (CTRL) and seven PAH subjects were serum deprived for 48 h and subjected to immunoblot analysis to detect indicated proteins. Immunoblots **(A)** and statistical analysis **(B)** are shown. Data are means ± SE, fold to control, *p < 0.05 by Mann–Whitney *U* test. **(C–G)** Early passage human PAH PAVSMC were treated with Akt inhibitor VIII (1, 5 μM) or vehicle (0) for 24 h followed by immunoblot analysis. Data are fold to vehicle-treated group, *n* = 5 subjects/group, *p < 0.05, **p < 0.01 by Kruskal–Wallis rank test with Dunn pairwise comparison *post hoc* test.

One of the key regulators of glucose and lipid metabolism is a serine/threonine kinase Akt (38–42), which stimulates glucose uptake and glycolysis (43) and promotes *de novo* lipid synthesis through sterol regulatory binding protein (SREBP)-dependent expression of lipogenic enzymes (43, 44). Accumulating evidence from multiple groups, including ours, demonstrate pathological role of Akt as a regulator of pulmonary vascular cell metabolism, proliferation, pulmonary vascular remodeling, and overall PH (10, 45, 46). This allowed us to hypothesize that Akt might coordinate glucose metabolism and *de novo* lipid synthesis supporting PAVSMC hyper-proliferation in PAH. In agreement with published studies reporting Akt activation in PAH (10, 32), we found that S473-Akt phosphorylation was significantly increased in human PAH PAVSMC (Figures 3A,B). Importantly, Akt inhibitor VIII suppressed activation of both key lipogenesis enzymes, ACLY and ACC; ACLY deactivation was detected by decrease in S455-ACLY phosphorylation (47), and ACC deactivation was detected by an increase in inhibitory S79-ACC (48) phosphorylation (Figures 3C–F and Supplementary Figures 3A,B), suggesting that Akt supports increased lipogenesis in human PAH PAVSMC. Moreover, Akt inhibitor suppressed phosphorylation of ribosomal protein S6 (Figures 3C,G), the main downstream effector of mTOR complex 1 (mTORC1), a known activator of cell growth, proliferation, and lipid biogenesis (49). In summary, these data demonstrate that *de novo* lipid synthesis in PAH PAVSMC is regulated by Akt.

### SIRT7 Up-Regulates Akt and Lipogenic Enzymes in Human PAH PAVSMC

**FIGURE 4:**
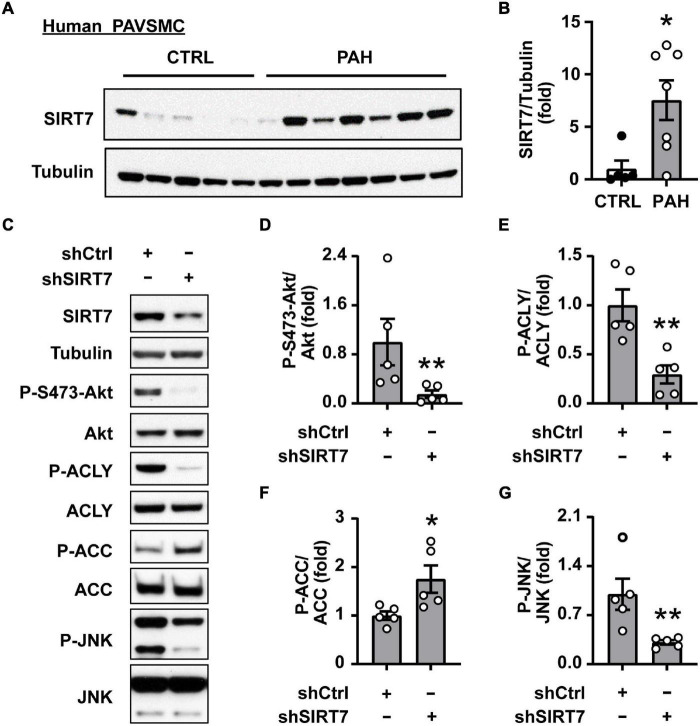
SIRT7 regulates Akt status and lipogenic enzymes activation in human PAH PAVSMC. **(A,B)** Early passage distal human PAVSMC from five non-diseased (CTRL) and seven PAH subjects were serum deprived for 48 h and subjected to immunoblot analysis to detect indicated proteins. Immunoblots **(A)** and statistical analysis **(B)** are shown. Data are means ± SE, fold to control. **(C–G)** Human PAH PAVSMC were transfected with shRNA SIRT7 (shSIRT7), or control scramble shRNA (shCtrl) for 72 h followed by immunoblot analysis. Data represent fold to shCtrl. Data are means ± SE, *n* = 5 subjects/group. *p < 0.05, **p < 0.01 by Mann–Whitney *U* test.

Next, we aimed to identify the factor(s) regulating *de novo* Akt-dependent lipid synthesis in PAH. Several studies demonstrate that lysine deacetylase SIRT7 coordinates cellular metabolic balance by regulating glucose sensing/homeostasis, glycolysis, mitochondria and ribosomal biogenesis, DNA damage response, fatty acid synthesis and overall lipid metabolism (50–56). Moreover, it was recently shown that SIRT7 modulates aortic vascular smooth muscle cell proliferation in wire injury model of the femoral artery (57) and promotes cancer progression by activating Akt (56, 58) and mTORC1 effector S6K1 (59). We found that SIRT7 protein levels were significantly higher in human PAH PAVSMC compared to non-diseased PAVSMC (Figures 4A,B). Importantly, shRNA-induced depletion of SIRT7 in PAH PAVSMC significantly reduced S473 Akt phosphorylation (Figures 4C,D) and activatory phosphorylation of ACLY, and significantly increased inhibitory phosphorylation of ACC in human PAH PAVSMC (Figures 4C,E,F), suggesting that Akt-mediated lipogenesis in human PAH PAVSMC is regulated by SIRT7.

### SIRT7-Dependent JNK Activation Is Required for Akt and Lipogenic Enzymes Up-Regulation in Human PAH PAVSMC

**FIGURE 5:**
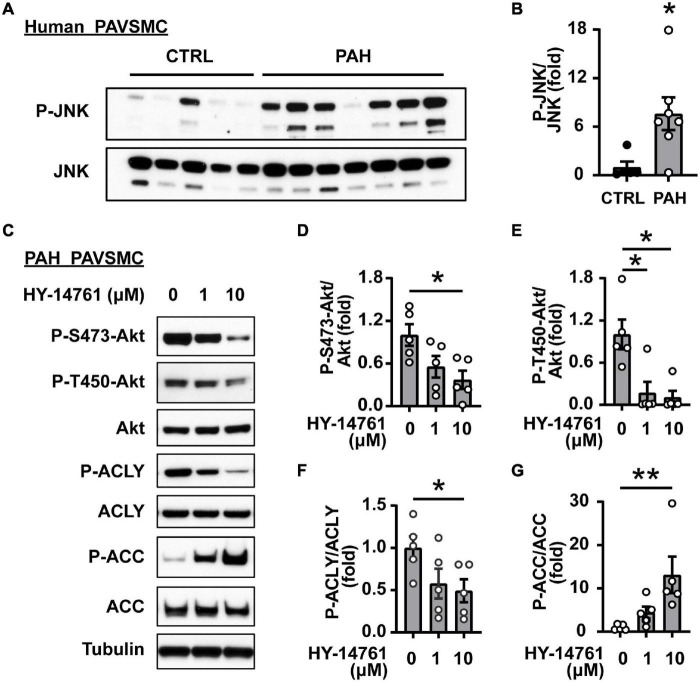
JNK regulates Akt and lipogenic enzymes in human PAH PAVSMC. **(A,B)** Early passage distal human PAVSMC from five non-diseased (CTRL) and seven PAH subjects were serum deprived for 48 h and subjected to immunoblot analysis to detect indicated proteins. Immunoblots **(A)** and statistical analysis **(B)** are shown. Data are means ± SE, fold to control, *p < 0.05 by Mann–Whitney *U* test. **(C–G)** Early passage human PAH PAVSMC were treated with JNK inhibitor HY-14761 (1, 10 μM) or vehicle (0) for 48 h followed by immunoblot analysis. Data represent fold to vehicle-treated group, *n* = 5 subjects/group, *p < 0.05, **p < 0.01 by Kruskal–Wallis rank test with Dunn pairwise comparison *post hoc* test.

Since SIRT7 does not possess kinase activity, its regulation of Akt phosphorylation status most likely is mediated by intermediate player(s) and one of the potential SIRT7 pro-survival targets is JNK (54). Indeed, we found that shSIRT7 significantly decreased phosphorylation of JNK in PAH PAVSMC (Figures 4C,G), also suggesting that SIRT7 regulates Akt and lipogenesis through JNK. Previous studies have shown that Akt activation *via* phosphorylation at S473 is achieved through a series of phosphorylation steps, and the initial priming phosphorylation of T450 can be regulated by JNK (60). We found a significant increase in T183/185 JNK phosphorylation in human PAH PAVSMC compared to control PAVSMC (Figures 5A,B), indicating that JNK is activated in PAH PAVSMC. JNK inhibitor HY-14761 significantly reduced both S473 and priming Thr450 Akt phosphorylation in PAH PAVSMC (Figures 5C–E). JNK inhibitor-mediated deactivation of Akt was associated with de-activation of lipogenic enzymes ACLY and ACC in human PAH PAVSMC (Figures 5C,F,G and Supplementary Figures 3C,D). These data indicate that Akt-mediated lipogenesis in human PAH PAVSMC is at least in part regulated by JNK.

In PAH, Akt activation in resident pulmonary vascular cells could be induced by excessive amounts of growth factors and pro-inflammatory mediators. To start determining pro-PH agents modulating SIRT7/JNK axis in PAVSMC, we treated non-diseased human PAVSMC with PDGF and IL-6. Interestingly, while both PGDF and IL-6 significantly increased JNK phosphorylation, only PDGF induced significant increase of SIRT7 protein levels (Supplementary Figures 4A,B), similar to those seen in human PAH PAVSMC. Together, these data suggest that SIRT7/JNK-dependent Akt activation in PAVSMC could be induced by PDGF.

### Akt Supports Lipid Accumulation, Proliferation and Survival of Human PAH PAVSMC

**FIGURE 6:**
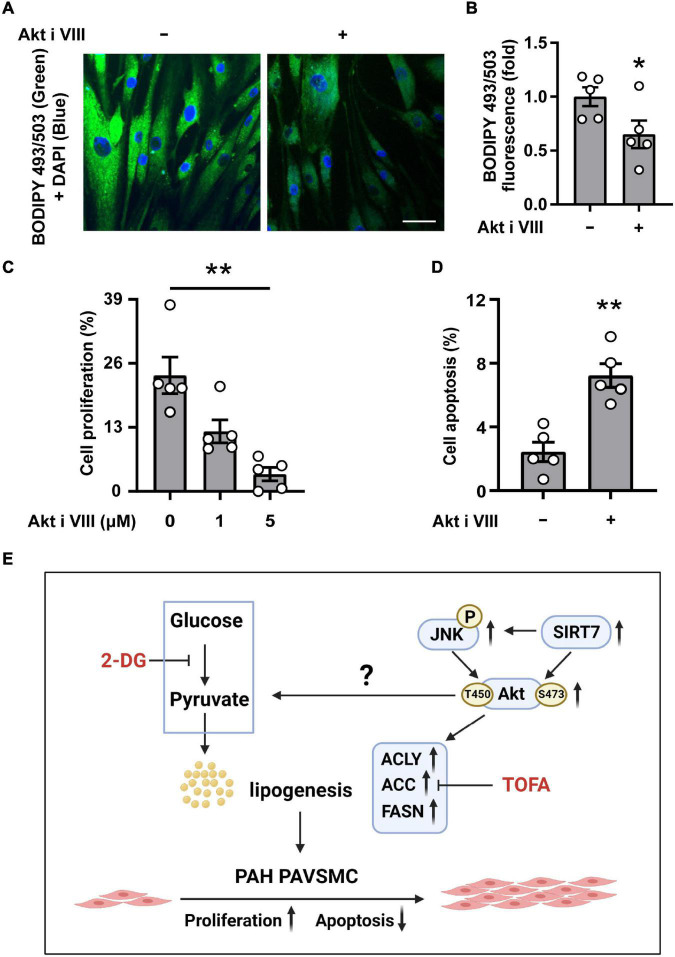
Akt supports intracellular lipid levels, proliferation and survival of human PAH PAVSMC. **(A,B)** Human PAH PAVSMC were incubated in serum deprived media supplemented with 0.1% lipid deprived BSA in presence of Akt inhibitor VIII (10 μM) or diluent. Forty-eight hours later neutral lipid accumulation was evaluated by fluorescent BODIPY 493/503 staining (green) followed by DAPI co-staining (blue) to detect nuclei. Representative images **(A)** and statistical analysis **(B)** are shown. Bar equals 50 μm. Data are means ± SE, fold to the diluent-treated cells, *n* = 5 subjects/group, *p < 0.05 by Mann–Whitney *U* test. **(C)** Human PAH PAVSMC were serum deprived in media, supplemented with regular 0.1% BSA, and were treated with 1, 5 μM **(C)** or 10 μM **(D)** Akt inhibitor VIII or vehicle (0/-) for 48 h followed by proliferation (Ki-67) **(C)** or apoptosis **(D)** analyses. Data are percentage of Ki-67- or TUNEL- positive cells/total number of cells, means ± SE, *n* = 5 subjects/group. *p < 0.05 Kruskal–Wallis rank test with Dunn pairwise comparison *post hoc* test **(C)** or by Mann–Whitney *U* test **(D)**. **(E)** Schematic representation of the proposed regulation of the *de novo* lipid synthesis, required for increased proliferation and survival of PAVSMC in PAH.

To further clarify the role of Akt in lipid accumulation, hyper-proliferation, and survival of human PAH PAVSMC, we treated cells with Akt inhibitor VIII. Immunocytochemical analysis demonstrated that intracellular lipid accumulation in PAH PAVSMC, maintained in lipid-deprived media, was significantly downregulated by Akt inhibitor VIII (Figures 6A,B). Moreover, treatment with Akt inhibitor VIII significantly reduced proliferation and promoted apoptosis of PAH PAVSMC (Figures 6C,D), demonstrating that Akt supports increased cell proliferation, survival, and lipogenesis in PAH PAVSMC (Figure 6E). Furthermore, inhibition of Akt did not augment 2-DG-dependent inhibition of PAH PAVSMC proliferation (Supplementary Figures 5A,B), suggesting that Akt regulates PAH PAVSMC proliferation at the level of or downstream of glycolytic enzymes. Taken together, our data demonstrate that human PAH PAVSMC have up-regulated lipogenesis supported in an Akt- and glycolysis-dependent manner to sustain increased proliferation and survival, and that Akt signaling is regulated, at least in part, by SIRT7-JNK axis (Figure 6E).

## Discussion

Increased proliferation and survival of PAVSMC in small PAs are critical components of pulmonary vascular remodeling in PAH, the mechanisms of which are not completely understood. Here we report that human PAH PAVSMC have up-regulated lipogenesis, which is required to support increased cell proliferation and survival. We also show that observed *de novo* lipid synthesis is glucose-dependent and is regulated by Akt. We further demonstrate that SIRT7 and JNK are up-regulated in PAH PAVSMC, and support Akt activation and lipogenesis. Lastly, we show that Akt activation is required for increased lipid accumulation, cell proliferation and survival of PAH PAVSMC. Overall, although further studies are needed, our data suggest that the SIRT7/JNK-Akt-lipogenesis axis could be considered as a potential target pathway for developing novel anti-remodeling therapy for PAH.

Metabolic alterations in glucose homeostasis and glycolysis, similar to the “Warburg effect” in cancer cells, support increased PAVSMC proliferation and pulmonary vascular remodeling in PAH (9, 10, 12). Increased glucose uptake coupled with unaltered fatty acid uptake by PAH PAVSMC (23) indicates that intracellular demand in lipids in these highly proliferative cells is fulfilled by alternative internal pathways. This phenomenon is well known in cancer, since tumor cells demonstrate elevated endogenous fatty acid synthesis, supported by the increased glycolysis, to maintain hyper-proliferation (29). Glucose serves as major source supporting lipid synthesis. Glucose is converted to pyruvate through glycolysis. Pyruvate, in turn, enters mitochondria for citrate generation. Through mitochondrial carrier Slc25a1, citrate can be exported into the cytosol, wherein it is cleaved by ACLY to produce acetyl-CoA, which is processed for *de novo* lipogenesis by ACC and FASN (61). We demonstrate that all the key enzymes of fatty acid synthesis, ACLY, ACC, and FASN are up-regulated in human PAH PAVSMC. Moreover, we found that PAH PAVSMC maintain hyper-proliferation and glucose-dependent accumulation of intracellular lipids even in the absence of an extracellular lipid source but require activation of intracellular *de novo* lipid synthesis machinery. This is an important observation, confirming that lipid synthesis plays a crucial role not only in RV disfunction in PAH (62, 63) but also in smooth muscle proliferation and pulmonary vascular remodeling. It also supports previous studies suggesting that targeting lipid metabolism in PAH VSM needs to be considered for developing on anti-remodeling therapeutic options for PAH treatment (27, 28).

Besides glucose, glutamine can also contribute carbon to lipogenic acetyl-coenzyme A (acetyl-CoA) through glutamine-derived α-ketoglutarate (α-KG). Generated α-KG could be, in turn, converted into citrate *via* an isocitrate dehydrogenase-1 (IDH1)-dependent mitochondrial or cytosolic pathway (64, 65). In our present study, we have shown that PAH PAVSMC *de novo* lipogenesis depends on glucose, but whether α-KG contributes to this process remains unstudied.

Akt signaling is tightly related to the regulation of glycolysis and lipogenesis (66–68). Akt stimulates aerobic glycolysis in cancer cells, supporting continued growth and survival, mediates bioenergetic stability in epithelial cells (68), and stimulates hepatic SREBP1c and lipogenesis through mTORC1-dependent and independent pathways (69), suggesting that Akt might be involved in regulation of lipid synthesis and accumulation in PAH PAVSMC. Our data demonstrate that Akt not only supports glycolysis-driven lipogenesis in PAH PAVSMC, but also regulates proliferation and survival of PAH PAVSMC. Since Akt is a major player, coordinating multiple processes directly involved in cell survival, growth, metabolism, proliferation, migration and differentiation (70), pharmacological targeting of Akt is a highly attractive therapeutic approach for proliferative diseases such as cancer. Multiple Akt inhibitors are now in various stages of clinical development (71, 72). However, since Akt activation occurs through various mechanisms, clinical efficacies of Akt inhibitors are limited (73), and Akt targeting still remains a challenge. Thus, inhibiting Akt *via* modulating its upstream regulators might represent an alternative, potentially attractive strategy for therapeutic intervention.

The members of the sirtuin family of lysine deacylases are important regulators of metabolic pathways and energy homeostasis (50, 74) which are also known regulators of Akt (75). Sirtuins have been implicated in multiple metabolic diseases, including aging, cancers, cardiovascular diseases, obesity, and diabetes mellitus (50) and are considered potential targets for the therapeutic interventions (76). Unlike other sirtuins, SIRT7 demonstrates a relatively weak deacetylase activity, but is involved in regulating cellular energy metabolism homeostasis, lipid metabolism, cell migration, and was recently shown to control VSMC proliferation (50, 57, 77, 78). SIRT7 is also considered an attractive target for anti-cancer therapy (79). Despite being the least well-characterized member of the sirtuin family, accumulating evidence shows that SIRT7 acts as an Akt upstream regulator in other cell types (58, 59, 80). Here we demonstrate that SIRT7 acts as an upstream positive regulator of Akt in PAH PAVSMC and supports Akt phosphorylation in a JNK-dependent manner. We found that pharmacological inhibition of JNK significantly reduced both S473 and T450 Akt phosphorylation in PAH PAVSMC. This finding is in agreement with previous studies, demonstrating that JNK regulates Akt reactivation through T450 phosphorylation in cardiomyocyte survival after hypoxia (60).

Our study, however, has limitations. Although use of cells from PAH and non-diseased human subjects strongly suggests the translational significance of our findings, we only verified the role of SIRT7-JNK-Akt-*de novo* lipid synthesis *in vitro*, and further studies are needed to determine the role of this axis *in vivo*. Also, further pre-clinical studies are needed to evaluate whether the link between SIRT7-JNK and Akt-dependent *de novo* lipid synthesis, required for increased proliferation and survival of PAH PAVSMC, could be a potential target pathway for therapeutic intervention.

In summary, we found that human PAH PAVSMC have up-regulated lipogenesis supported in an Akt- and glycolysis-dependent manner to sustain increased cell proliferation. We also show that inhibition of the Akt-lipogenesis axis reduces proliferation and induces apoptosis of human PAH PAVSMC. In aggregate, our data provide a link between glycolysis, lipogenesis and proliferation of human PAH PAVSMC and call for further studies to determine the potential attractiveness of the SIRT7/JNK-Akt-lipogenesis axis as a target pathway for therapeutic intervention.

## Data Availability Statement

The data that support the findings of this study are available from the corresponding author upon reasonable request.

## Author Contributions

EG and TK: conception and design. LJ, TK, HD, EG, DG, AP, BC, YS, and DL: experimental work, analysis, and interpretation. LJ, EG, and TK: drafting the manuscript and intellectual content. All authors contributed to the article and approved the submitted version.

## Conflict of Interest

The authors declare that the research was conducted in the absence of any commercial or financial relationships that could be construed as a potential conflict of interest.

## Publisher’s Note

All claims expressed in this article are solely those of the authors and do not necessarily represent those of their affiliated organizations, or those of the publisher, the editors and the reviewers. Any product that may be evaluated in this article, or claim that may be made by its manufacturer, is not guaranteed or endorsed by the publisher.

## Supplementary Material

Supplementary Figure 1 **(A,B)** Early passage distal primary human PAVSMC from non-diseased (CTRL) and PAH subjects were serum-deprived for 48 h and subjected to immunoblot analysis to detect indicated proteins. **(A)** *n* = 5 (CTRL), *n* = 7 (PAH) (see Figure 1B for statistical analysis). **(B)** Data are means ± SE, fold to control, *n* = 5 subjects/group.

Supplementary Figure 2 Early passage PAVSMC from five non-diseased (CTRL) and seven PAH subjects were serum deprived for 48 h and subjected to immunoblot analysis to detect indicated proteins. Immunoblots **(A)** and statistical analysis **(B)** are shown. **(B)** Data are means ± SE, fold to control.

Supplementary Figure 3 Early passage human PAH PAVSMC were treated with Akt inhibitor VIII, JNK inhibitor HY-14761, or diluent (0) and subjected to immunoblot analysis to detect indicated proteins. Data are fold to diluent-treated group. **p* < 0.05 by Kruskal–Wallis rank test with Dunn pairwise comparison *post hoc* test.

Supplementary Figure 4 Early passage human control (non-diseased) PAVSMC were treated with **(A)** PDGF-BB (60 ng/ml) or **(B)** IL-6 (20 ng/ml) and appropriate vehicles for 48 h followed by immunoblot analyses of indicated proteins. Representative immunoblots and statistical analyses are shown. Data are means ± SE, *n* = 3 subjects/group, **p* < 0.05, by Mann–Whitney *U* test.

Supplementary Figure 5 Early passage human PAH PAVSMC were incubated in serum deprived media in presence of 2-Deoxy-D-glucose (2-DG) 10 mM **(A)** or 1 mM **(B)** and/or Akt inhibitor VIII (1 μM) or vehicle (−). Forty-eight hours later cell proliferation (Ki-67) was analyzed. Data are means ± SE, percentage of Ki-67-positive cells/total. **p* < 0.05, ***p* < 0.01 by Kruskal–Wallis rank test with Dunn pairwise comparison *post hoc* test.
