# Supplementary material for: Akt-Dependent Glycolysis-Driven Lipogenesis Supports Proliferation and Survival of Human Pulmonary Arterial Smooth Muscle Cells in Pulmonary Hypertension
Source: Front Med (Lausanne). 2022 Jun 28;9:886868. doi: 10.3389/fmed.2022.886868 (PMC9274086; doi:10.3389/fmed.2022.886868)

Supplementary Figure 1

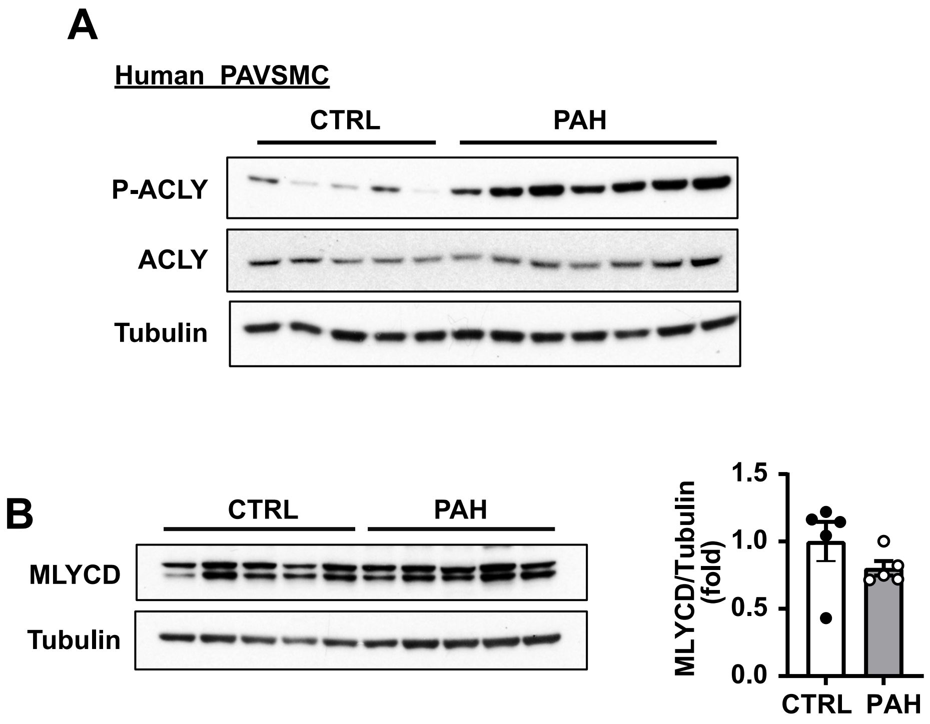

Supplementary Figure 2

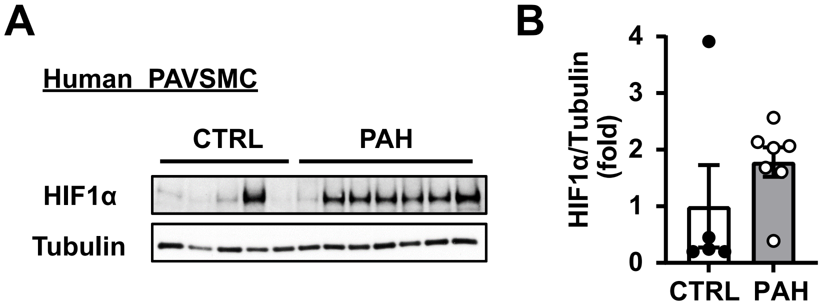

Supplementary Figure 3

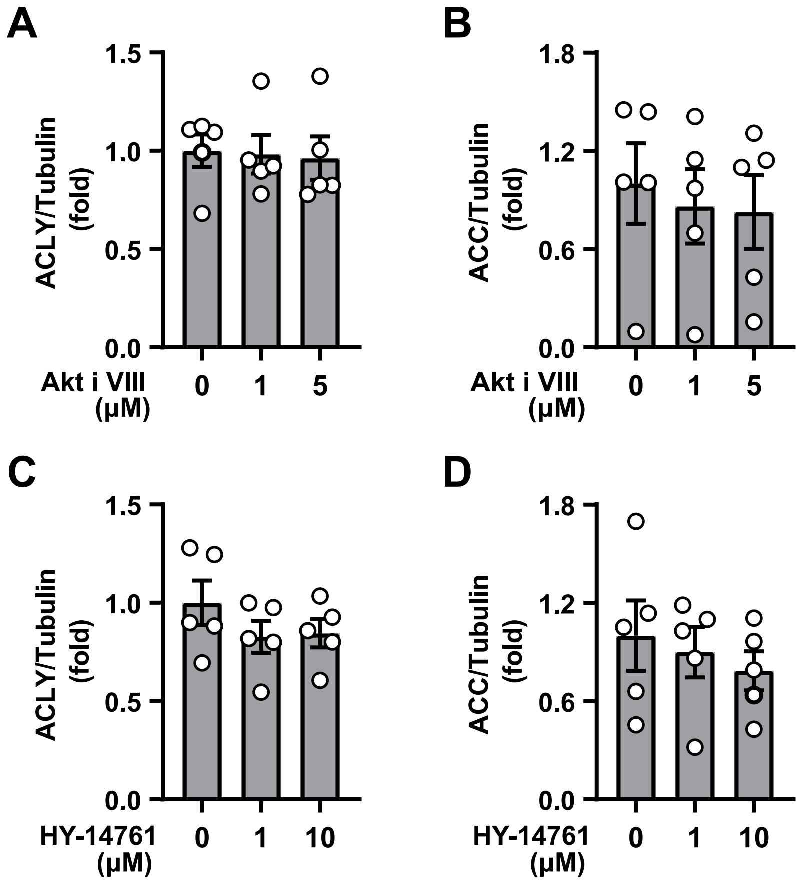

Supplementary Figure 4

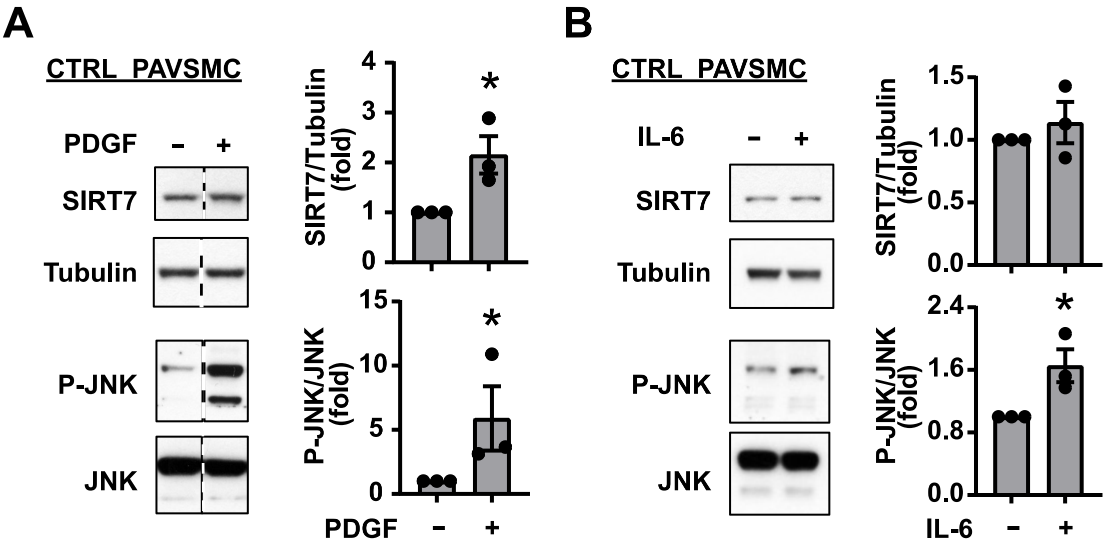

Supplementary Figure 5

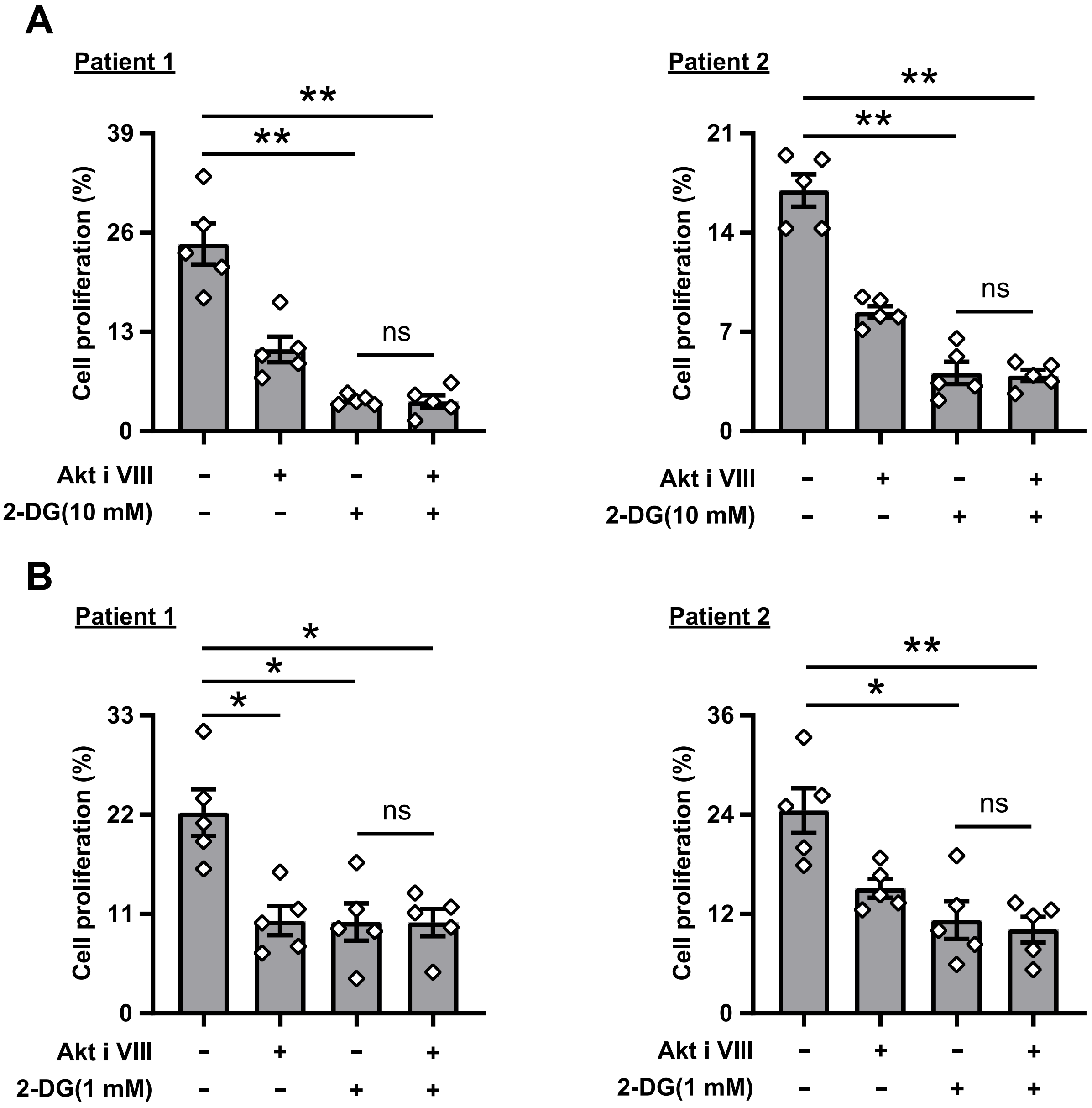

Supplement: Supplementary Figure 1 — (A,B) Early passage distal primary human PAVSMC from non-diseased (CTRL) and PAH subjects were serum-deprived for 48 h and subjected to immunoblot analysis to detect indicated proteins. (A) n = 5 (CTRL), n = 7 (PAH) (see Figure 1B for statistical analysis). (B) Data are means ± SE, fold to control, n = 5 subjects/group. [file Data_Sheet_1.pdf]
